# The Impact of Selective Dry Cow Therapy Adopted in a Brazilian Farm on Bacterial Diversity and the Abundance of Quarter Milk

**DOI:** 10.3390/vetsci9100550

**Published:** 2022-10-08

**Authors:** Juliano L. Goncalves, Juliana Young, Renata de F. Leite, Carlos E. Fidelis, Priscila A. Trevisoli, Luiz L. Coutinho, Nathália C. C. Silva, Roger I. Cue, Vera Lucia Mores Rall, Marcos V. dos Santos

**Affiliations:** 1 Department of Animal Nutrition and Production, School of Veterinary Medicine and Animal Sciences, University of São Paulo (USP), Pirassununga, São Paulo 13635-900, Brazil; 2 Department of Large Animal Clinical Sciences, College of Veterinary Medicine, Michigan State University (MSU), East Lansing, MI 48864, USA; 3 Department of Bacteriology, University of Wisconsin-Madison (UW), Madison, WI 53706, USA; 4 Luiz de Queiroz College of Agriculture, University of São Paulo (USP), Piracicaba, São Paulo 13418-900, Brazil; 5 Department of Food Science and nutrition, Faculty of Food Engineering, University of Campinas, Campinas, São Paulo 13083-862, Brazil; 6 Department of Animal Science, Macdonald Campus, McGill University, Quebec, QC H9X 3V9, Canada; 7 Department of Chemical and Biological Sciences, Institute of Biosciences, Sao Paulo State University, Botucatu, São Paulo 18618-689, Brazil

**Keywords:** antibiotic resistance, selective dry cow therapy, internal teat sealant, bacterial diversity, new intramammary infection

## Abstract

**Simple Summary:**

The current study sought to assess the impact of selective dry cow therapy (SDCT) (protocol 1: antibiotics combined with internal teat sealant (ITS); vs. protocol 2: ITS alone) on bacterial diversity and the abundance of quarter milk. Based on the results of bacteriological culturing, the quarters (*n* = 313) were categorized as healthy, cured, persistent, and new intramammary infection. The bacterial diversity was similar when comparing both healthy and cured quarters submitted to both drying-off protocols. Although healthy cows that were treated at drying-off using only teat sealant showed no alteration in the alpha and beta diversity of bacteria, they showed a higher abundance of bacterial groups that may be beneficial to or commensals of the mammary gland, which implies that antibiotic therapy should be reserved for mammary quarters with a history of mastitis.

**Abstract:**

We aimed to evaluate the impact of selective dry cow therapy (SDCT) (protocol 1: antimicrobial combined with internal teat sealant (ITS); vs. protocol 2: ITS alone) on bacterial diversity and the abundance of quarter milk. Eighty high production cows (parity ≤ 3 and an average milk yield of 36.5 kg/cow/day) from the largest Brazilian dairy herd available were randomly selected; milk quarter samples were collected for microbiological culture (MC) on the day of drying-off (*n* = 313) and on day 7 post-calving (*n* = 313). Based on the results of the MC before and after calving, 240 quarters out of 313 were considered healthy, 38 were cured, 29 showed new infections and 6 had persistent infections. Mammary quarters were randomly selected based on intramammary information status and SDCT protocols for bacterial diversity analyses. The bacterial diversity was similar when comparing both healthy and cured quarters submitted to both drying-off protocols. Despite healthy cows that were treated at dry-off using only teat sealant showing no alteration in the alpha and beta bacterial diversity, they did show a higher abundance of bacterial groups that may be beneficial to or commensals of the mammary gland, which implies that antibiotic therapy should be reserved for mammary quarters with a history of mastitis.

## 1. Introduction

Dry cow therapy (DCT) refers to the intramammary administration of antimicrobials, which can be performed either in association with internal teat sealant (ITS) or not, to cure intramammary infections (IMI) acquired during lactation and to prevent the occurrence of new intramammary infections (NIMI) during the dry period [1,2]. Using DCT in all cows (also known as blanket DCT) results in the use of antibiotics (ATB) in healthy cows. Over 90 percent of dairy herds in the United States use blanket DCT to control udder health during the dry period [3]. Additionally, the application of blanket DCT translates to the use of 11 tons of ATB per year in the United States [3]. Aside from the increased cost and risk of antimicrobial resistance (AMR), using ATB in healthy cows during the dry period increases the risk of subclinical mastitis [4].

The non-prudent use of antibiotics in production animals can reduce the active ingredient’s effectiveness, consequently increasing the development of AMR. AMR can lead to a decreased availability of ATBs, limiting the ability to control diseases in animal production [5]. A global plan of action on AMR was recently established at the World Health Assembly [6]. The plan emphasizes the importance of a coordinated “one-health” approach involving numerous sectors and international actors, including human and veterinary medicine, agriculture, finance, the environment, and consumers. Considering global concerns regarding AMR, selective dry cow therapy (SDCT) is an alternative way to reduce the use of ATB in dairy herds. According to recent research, SDCT, based on on-farm culture results and ITS usage, can reduce ATB use without increasing NIMI and somatic cell count (SCC) in the subsequent lactation [7,8,9]. Furthermore, a thorough understanding of mastitis mechanisms is critical for promoting animal health while also encouraging the assertive use of ATB in dairy cows. Thus, prior to performing blanket DCT, identifying healthy cows can be an alternative way of reducing the use of ATB.

To encourage the prudent use of ATB, and as an alternative to blanket DCT, the use of ITS alone has been studied as an alternative to prevent NIMI during the dry period [10,11]. ITS are inert substances that are usually based on bismuth subnitrate, which serves as a physical barrier in the teat cistern, mimicking the physiological keratin and preventing microbial access via the teat canal. This technology can be used in addition to or instead of DCT in the prevention of NIMI during the dry period [8,12]. ITS are not absorbed after intramammary infusion, remaining in the teat canal throughout the dry period, until removal in the first milking or during the subsequent lactation [13,14]. In a study evaluating the use of ITS in the prevention of NIMI, an efficacy similar to that of DCT (ATB-based) was observed [13]. Compared to untreated quarters, the use of ITS reduced the number of NIMI in the post-calving period and the occurrence of clinical mastitis in the first 100 days of the subsequent lactation [15].

SDCT is based on the use of ATB + ITS in cows with a history of high SCC and/or microorganism isolation, based on on-farm culture results, while healthy cows have been treated only with ITS because of the criteria of low SCC and no clinical mastitis history, and/or no pathogen isolation using on-farm culture. Through the use of this alternative selective protocol, antimicrobials for drying-off can be reduced by 55% while still ensuring that the health of dairy cows’ mammary glands is protected [16].

Studies on bacterial diversity in milk are scarce during the drying-off period [17,18]. In a recent study, the incidence of bacterial load and bacterial diversity in milk from healthy cows when using ITS was comparable to that seen in cows receiving DCT (ATB + ITS), but only healthy cows were included in the experimental design [11]. In fact, some microorganisms can be part of the milk microbiome without growing on blood agar, as they are not cultivable by the standard identification methodology. Possibly, parts of these bacterial groups may be beneficial or commensals of the mammary gland. Thus, the use of DCT in healthy quarters could affect part of the ‘beneficial’ microbiome and favor the increase of AMR because of the non-assertive use of ATB. In this context, the present study offers the general hypothesis that SDCT positively affects the management of healthy cows, since the adoption of ITS alone would reduce the use of ATB, thus, not altering the bacterial diversity of milk. The purpose of this study was to compare the impact of SDCT (protocol 1: ATB combined with ITS vs. protocol 2: ITS alone) on bacterial diversity and the abundance of quarter milk.

## 2. Materials and Methods

### 2.1. Herd Selection and Cow Enrollment

The research was carried out from December 2017 to July 2018 on the biggest dairy herd of Sao Paulo State, Brazil. At the start of the study, the average number of lactating cows in the herd was 1850, with a milk production of 36.4 kg/cow/day ± 6.2 L (mean ± SD). The herd was selected based on (i) the farmer’s willingness to participate and the regular use of SDCT, (ii) having an availability of data-recording systems, and (iii) routine diagnosis of clinical mastitis. Cows were housed in free stall barns during lactation, while during the dry period, all cows were kept in open paddocks, received water ad libitum, and were fed in accordance with the herd’s dietary management program.

All the selected Holstein cows in this study (*n* = 80; 1.9 ± 0.8 (the number of cows lactating); parity ≤ 3) were in late gestation (347 days ± 90), were clinically healthy (no signs of clinical mastitis) and did not receive any antibiotics or anti-inflammatory drugs 30 days before drying-off. The cows’ expected dry period lengths ranged from 45 to 80 days. On the day of drying-off (*n* = 313) and on day 7 post-calving (*n* = 313), sterile milk samples were obtained at the mammary quarter level for microbiological culture (MC) analysis, in accordance with the National Mastitis Council (NMC) recommendations (2017) [19].

### 2.2. Farm Protocols for Treatment and Milk Sampling

The random assignment of cows at the drying-off period was based on the criteria of the farm: (a) healthy, when all four quarters had no isolation of mastitis-causing agents, no history of clinical mastitis, and the last three monthly DHI tests were SCC < 200 × 10^3^ cells/mL, which were only treated with 2.6 g bismuth subnitrate ITS; and (b) subclinical mastitis, culture-positive in at least one quarter, and a minimum in one of the three monthly SCCs of > 200 × 10^3^ cells/mL), which were treated with 250 mg cephalonium and ITS.

For SDCT, intramammary ATB was injected into each teat, then massaged vertically. Following the infusion of ATB, each teat received ITS and was then submerged in a commercial iodine-based teat dip (1% iodine, Della Barrier, DeLaval, Tumba, Sweden). To ensure that the ITS stayed in the teat canal, the teat base was held with two fingers and no massage was given, as described by previous researchers [2]. Farm personnel administered both protocols. After treatment administration, milking was stopped abruptly.

### 2.3. Conventional Microbiology and Species Confirmation by MALDI-TOF MS

A 10-μL sample of milk was streaked onto 5% bovine blood agar and incubated at 37 °C for 48 h. After incubation, plates were examined for colony morphology once every 24 h (i.e., colony number, size, pigmentation, and hemolysis) [19]. Samples with the growth of over two different colonies were considered contaminated. The species-level identification of pathogens was conducted using MALDI-TOF MS [20]. In short, a wooden applicator stick was used to place one colony on the steel-plate spot. The spot was treated with 1.0 μL of 70% formic acid. After drying at room temperature, 1.0 μL of α-cyano-4-hydroxycinnamic acid matrix solution was used in each spot. The 96-spot plate was allowed to dry for 5 to 10 min at room temperature. The bacterial test standard was used for MALDI-TOF MS calibration. The analysis was carried out using FlexControl 3.4 software; each measurement was subjected to the identification score cut-off values in the MALDI Biotyper system, with a score of ≥ 2 indicating a species-level identification (Bruker Daltonik, Billerica, MA, USA).

### 2.4. Mammary Gland Health Indicators Definition

Based on the obtained results of MC performed at the laboratory, four intramammary infection categories were considered and distributed by the two protocols of treatment: healthy (no isolation of microorganisms in all microbiological culture); cured (a positive isolation result on the day of drying-off, but a negative result after calving); persistent (when the same microorganisms were isolated on the day of drying-off and after calving); new infection (no isolation of microorganisms on the day of drying-off, but a positive isolation result after calving, or if the microbiological culture result after calving differed from the one on the day of drying-off).

### 2.5. DNA Extraction, Library Preparation, and Sequencing

A total of twenty mammary quarters from twenty cows were randomly selected based on cow treatment (DCT + ITS vs. ITS alone) and intramammary infection categories (healthy, cured, NIMI, and persistent). The selected quarters had milk sampled three times, at the drying-off (day 0), post-calving (day 7), and an additional sampling at day 14 post-calving, for MC and next-generation sequencing (NGS) (Illumina Inc., San Diego, CA, USA). This study was primarily concerned with the detection of bacterial communities and used primers designed specifically for 16S rRNA amplification. For DNA extraction, 2 mL of milk was put into a 2 mL microcentrifuge tube and spun at 14,000 rpm for 5 min. After centrifugation, the fat and supernatant were thrown away and the pellet was kept at −20 °C for further DNA extraction. Genomic DNA was obtained using a MagMAX™ CORE, combined with the MagMAX™ CORE mechanical lysis module (Thermo Fisher™) in accordance with the manufacturer’s guidelines. Library preparation was performed according to 16S Metagenomic Sequencing Library Preparation Guidelines (Illumina Inc., San Diego, CA, USA). The primers 515F (5′ GTGYCAGCMGCCGCGGTAA 3′) and 806BR (5′ GGACTACNVGGGTWTCTAAT 3′) were used to amplify the V4 hypervariable region from the 16S rRNA gene by PCR. Equimolar quantities of each library were pooled, and sequencing was performed using MiniSeq High Output reagent kit (300 cycles) on the MiniSeq platform (Illumina Inc., San Diego, CA, USA). All DNA sequences have been submitted to the NCBI’s Short Read Archive as part of BioProject PRJNA776327.

### 2.6. Bioinformatics Analysis

DNA sequences were analyzed using mothur (v. 1.39.0) [21] following the MiSeq SOP pipeline with modifications for Amplicon Sequence Variant (ASV) inference (https://mothur.org/wiki/miseq_sop/#asvs; accessed on 10 July 2021). In brief, paired-end reads were joined using the default parameters in ‘make.contigs’, and sequences with lengths of less than 200 bp or that were greater than 500 bp that contained ambiguous characters or had a homopolymer greater than 8 bp were removed. The remaining sequences were aligned against SILVA 16S rRNA gene reference database (release 138) [22], and then pre-clustered for ASV inference and error-correction of amplicon reads via UNOISE3 algorithm [23]. Sequences were chimera-filtered via Uchime algorithm [24], and then taxonomically assigned using SILVA 138 reference database [22] with a consensus confidence threshold of 80% [25]. Any sequences that did not align to the right region, that were chimeric and or classified as archaea, eukaryote, cyanobacteria, chloroplasts, and mitochondria were removed. To address different sequencing depths, the ASV table was normalized by the method total group in which sequences were subsampled to the number of sequences in our smallest group and then normalized across samples to produce equal sequence counts (9309 sequences per sample). The normalized ASV table was used to calculate alpha diversity indices including the number of observed ASVs (Observed), Shannon index-based measure of evenness (Shannon) [26], and the inverse Simpson index-based measure of diversity (Invsimpson) [27]. Beta diversity was also determined using the Bray-Curtis dissimilarity index [28], as well as the relative abundance (reads/total reads in a sample × 100) of ASVs in each sample. Alpha diversity indices were obtained via mothur (v1.39.0), whereas the Bray-Curtis dissimilarity index was calculated using the function vegdist, available in the vegan R package (v2.5-6) [29].

### 2.7. Statistical Analysis

All statistical analyses were performed in R (v.4.1.1, R Core Team, 2021) and tests were assessed as significant if the *p*-values and/or false discovery rate (FDR) ≤ 0.05. Measurements of the alpha diversity (Observed, Invsimpson and Shannon), as well as the relative abundance of ASVs, were assessed for normality and were found to follow a nonnormal distribution. Thus, differences in the bacterial alpha-diversity of milk samples from healthy and cured cows in response to treatment (DCT + ITS vs. ITS), time of sampling (day 0: the drying-off day, and on days 7 and 14 post-calving), and the interaction between these two factors were assessed under gamma distribution using a repeated-measures generalized linear mixed model, estimated via penalized quasi-likelihood. The resulting ANOVA *p* values were adjusted for false-discovery rate (FDR) using the Benjamini–Hochberg method. In the presence of significant effects, multiple comparisons among the least-squares means (LSMEANS) were performed with a SIDAK adjustment. These analyses were performed using functions available in the R package: MASS (v7.3-51.5), LSMEANS (v2.30-0), and ggplot2 (v3.2.1) [30,31].

To visually explore the degree of dissimilarity between the bacterial composition of milk samples from healthy and cured cows, treated with DCT + ITS or ITS alone, and sampled at three distinct time points (day 0: the drying-off day and on days 7 and 14 post-calving), principal coordinate analysis (PCoA) was conducted on the Bray–Curtis distance matrix [28]. In addition, a permutational multivariate analysis of variance (PERMANOVA; nperm = 1000) was carried out to evaluate the differences in the composition of bacterial communities, according to treatment, time of sampling, and the interaction of these factors within each category (healthy and cured). These analyses were performed with ASVs present in at least 80% of all samples using the functions available in the R packages vegan (v2.5-6) and ggplot2 (v3.2.1)

Lastly, we sought to determine if the abundance of bacterial ASV, summed to genus level, was differentially abundant between treatments (DCT + ITS vs. ITS), as well as times of sampling (day 0: the drying-off day, and on days 7 and 14 post-calving). To this end, we employed an ANCOM-BC (analysis of compositions of microbiomes with bias correction) model that considers the compositional nature of amplicon sequencing data and corrects the bias induced by differential sampling fractions across samples [32]. Only bacterial ASVs that were detected in at least 80% of all samples were included. The Benjamini–Hochberg method was used to adjust the *p*-values for FDR. This analysis was carried out using functions from the ANCOM-BC package, and the results were plotted using ggplot2 [29,33].

## 3. Results

### 3.1. Frequency of Mastitis-Causing Pathogens on the Day of Drying-off and Day 7 after Calving

Based on the results of MC, 240 quarters out of 313 were considered healthy (76.7%), 38 cured (12.1%), 29 NIMI (9.3%), and 6 persistent (1.9%). The most frequently isolated microorganisms during drying-off were non-aureus Staphylococci (52.4%), *Corynebacterium* spp. (19%), Enterobacteriaceae sp. (14.3%), *Prototheca* spp. (4.8%), *Streptococcus dysgalactiae* (2.4%), *Streptococcus uberis* (2.4%), *Trueperella pyogenes* (2.4%), and *Lactococcus garvieae* (2.4%).

Of the total 313 sampled quarters on the day of SDCT, 86.6% had culture-negative results. From the remaining sampled quarters after calving, 88.8% had culture-negative results. Of a total of sixty milk mammary quarters samples that were submitted to NGS, culture results, treatment groups for the adoption of drying-off protocols, and intramammary infection categories definition are presented in Table 1.

### 3.2. Alpha and Beta Diversity Analysis

**Figure 1:**
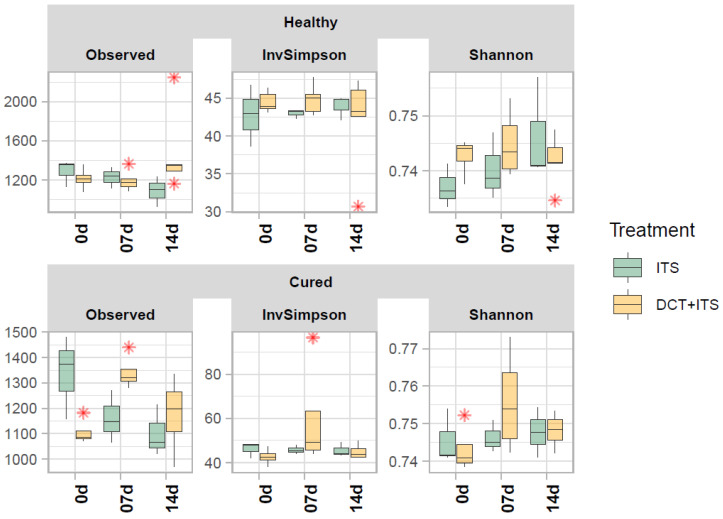
Alpha diversity of bacterial communities of the milk samples. Boxplots demonstrate the distribution of alpha diversity indices: number of observed ASVs (Observed), Shannon’s evenness (Shannon) and inverse of Simpson’s diversity (Invsimpson) for each sample grouped according to category (Healthy and Cured), treatment (ITS and DCT+ITS), and time of sampling (0 d: the day of drying-off, 7 d: 7 days after calving, and 14 d: 14 days after calving). Boxes represent the interquartile range (IQR) between the first(25th) and third quartiles (75th percentiles) whereas the horizontal line represents the median. Whiskers represent the lowest and highest values within 1.5 times the IQR from the first and third quartiles, respectively. The “ * ” represent outliers.

**Figure 2:**
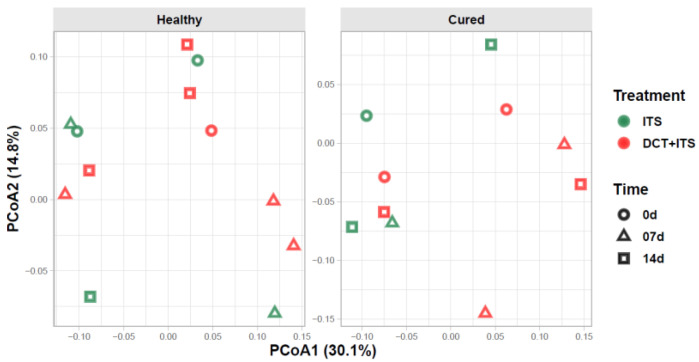
Beta diversity of bacterial communities of the milk samples. Principal coordinate analysis (PCoA) showing the Bray-Curtis dissimilarities in the composition of bacterial communities between milk sample grouped according to category (Healthy and Cured), treatment (ITS and DCT+ITS), and time of sampling (0 d: the day of drying-off, 7 d: 7 days after calving, and 14 d: 14 days after calving). Individual points in each plot represent a milk sample, while different colors and shapes represent treatments and time of sampling, respectively. Percentages shown along the axes represent the proportion of dissimilarities captured by PCoA in a two-dimensional (2D) coordinate space.

In our bacterial alpha-diversity analysis of healthy and cured dairy cows, the number of observed ASV (richness) and the inverse of Simpson (diversity) and Shannon (evenness) did not vary significantly in response to treatment, time of sampling, or the interaction of these factors (Table 2, Table 3 and Table 4 and Figure 1). Similarly, our beta-diversity analysis showed that Bray–Curtis dissimilarities in the bacterial community of milk samples from healthy and cured cows were not significantly ascribed to treatment, time of sampling, or interaction of these factors, respectively (PERMANOVA, healthy: *p* = 0.97, 0.75 and 0.34; and cured: *p* = 0.15, 0.41 and 0.58, respectively; Table 5). Indeed, our PCoA analysis (Figure 2) showed that milk samples from healthy and cured cows treated only with ITS or with DCT + ITS and collected at 0 d, 7 d, and 14 d did not cluster together, suggesting that there is not a clear distinction in structure/composition in the bacterial communities between these groups.

### 3.3. Taxonomic Composition

Our taxonomic composition analysis of the bacterial communities in milk samples revealed a total of 51,128 unique ASVs assigned to 36 phyla, 352 families, or 634 genera. The average percentages of sequences unassigned to any phylum, family, or genus were 0.24% ± 0.01, 30.24% ± 0.34, and 50.23% ± 0.58, respectively. In summary, the most abundant and prevalent phyla present in at least 80% of all samples were Proteobacteria (62.24% ± 0.33), Planctomycetota (11.10% ± 4.20), Firmicutes (11.02% ± 0.13), Acidobacteriota (5.00% ± 0.63) and Actinobacteriota (4.36% ± 0.14). Lastly, the most abundant families were the Pseudomonadaceae (25.28% ± 0.42%), Gemmataceae (9.64% ± 5.51), Enterobacteriaceae (6.09% ± 0.52), Streptococcaceae (3.06% ± 0.09), and Bryobacteraceae (3.05% ± 1.99).

### 3.4. Differential Abundance Analysis

While the variations in the alpha and beta diversity of bacterial communities of milk samples from healthy and cured cows were not significantly ascribed to treatments (DCT + ITS or ITS), as well as the time of sampling (0 d, 7 d, and 14 d), our differential abundance analysis showed that the abundance of several taxa varied significantly and simultaneously with these two factors (ANCOM-BC, FDR ≤ 0.05).

**Figure 3:**
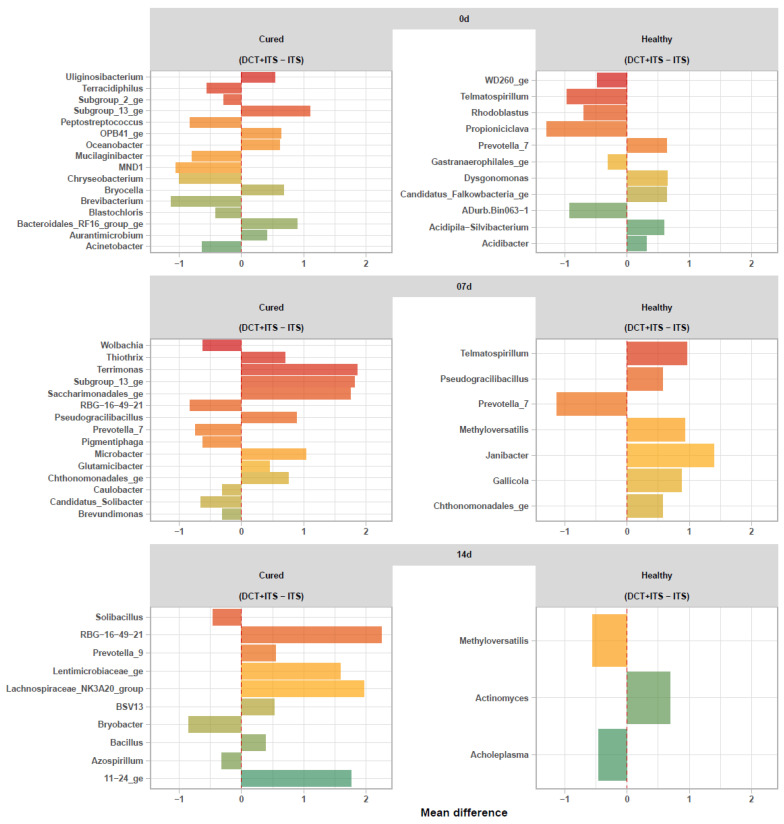
Differential abundance analysis of bacterial communities (ASVs summarized to genus level) of the milk samples. Waterfall plot showing significant changes (ANCOM-BC, FDR ≤0.05) in the abundance of bacterial genera in milk samples from DCT + ITS vs ITS within each sampling time (0 d: the day of drying-off, 7 d: 7 days after calving, and 14 d: 14 days after calving) and category (Healthy and Cured).

In general, milk samples collected on the day of drying-off (0 d) from healthy quarters of the antibiotic group (DCT + ITS) showed a significant decrease in *Propioniciclava*, *Telmatospirillum*, ADurb.Bin063-1, *Rhodoblastus*, WD260_ge and *Gastranaerophilales*_ge, but an increase in *Acidibacter*, *Acidipila-Silvibacterium*, *Prevotella*_7, *Candidatus_Falkowbacteria*_ge, and *Dysgonomonas* in comparison to quarters treated only with ITS. After calving, milk samples collected from healthy quarters on day 7 treated with (DCT+ITS) showed a significant decrease of *Prevotella*_7, but an increase of *Chthonomonadales*_ge, *Pseudogracilibacillus*, *Gallicola*, *Methyloversatilis*, *Telmatospirillum*, and *Janibacter* in comparison to quarters treated only with ITS. Lastly, milk samples collected from healthy quarters on day 14 after calving treated with (DCT + ITS) showed a significant decrease in *Methyloversatilis* and *Acholeplasma*, but an increase in Actinomyces, in comparison to quarters treated only with ITS (Figure 3).

Overall, milk samples collected on the day of drying-off (0 d) from cured quarters of the antibiotic group (DCT + ITS) showed a significant decrease in *Brevibacterium*, MND1, *Chryseobacterium*, *Peptostreptococcus*, *Mucilaginibacter*, *Acinetobacter*, *Terracidiphilus*, *Blastochloris*, and Subgroup_2_ge, but an increase in *Aurantimicrobium*, *Uliginosibacterium*, *Oceanobacter*, OPB41_ge, *Bryocella*, *Bacteroidales*_RF16_group_ge and Subgroup_13_ge *Actinomyces*, in comparison to quarters treated only with ITS. After calving, milk samples collected from cured quarters on day 7 and treated with (DCT + ITS) showed a significant decrease in RBG-16-49-21, *Prevotella*_7, *Candidatus_Solibacter*, *Pigmentiphaga*, *Wolbachia*, *Caulobacter*, and *Brevundimonas*, but an increase in *Glutamicibacter*, *Thiothrix*, *Chthonomonadales*_ge, *Pseudogracilibacillus*, *Microbacter*, *Saccharimonadales*_ge, Subgroup_13_ge, and *Terrimonas* in comparison to quarters treated only with ITS. Finally, milk samples collected from cured quarters on day 14 after calving and treated with (DCT + ITS) showed a significant decrease in *Bryobacter*, *Solibacillus*, and *Azospirillum* but an increase in *Bacillus*, BSV13, *Prevotella*_9, *Lentimicrobiaceae*_ge, 11–24_ge, the *Lachnospiraceae*_NK3A20_group and RBG-16-49-21, in comparison to quarters treated only with ITS (Figure 3).

## 4. Discussion

AMR is regarded as one of the world’s most critical public health threats, as the emergence of resistant bacterial pathogens can have disastrous consequences for human and animal health, as well as the global economy [34]. According to the World Health Organization (WHO), AMR might result in up to 10 million deaths and a cost of USD 100 trillion by 2050 [6]. Most antimicrobials are used in veterinary medicine to treat mastitis, an inflammation of the mammary gland caused by the immune system recognizing intramammary infection (IMI) that is mainly caused by bacterial pathogens. Thus, the non-prudent use of antimicrobials in human and veterinary medicine is considered to be a contributing driver to the emergence of AMR. In this context, we wondered whether SDCT would positively impact the management of healthy cows. In this way, the adoption of ITS alone could reduce the use of ATB without increasing NIMI and SCC, while not altering the bacterial diversity of milk. Therefore, this study aimed to evaluate the effect of SDCT (protocol 1: DCT + ITS, vs. protocol 2: ITS) on bacterial diversity and its abundance in quarter milk. Overall, our results suggested that the use of ITS alone is an excellent strategy for the rational use of ATB.

A high percentage of minor pathogens (non-aureus staphylococci (NAS) and *Corynebacterium* spp.) has been isolated in previous studies [35,36], as similarly found in the present study. However, it is still a matter of discussion whether these groups of pathogens cause true IMI since a lower mean SCC was reported in most cases when compared with healthy quarters [37,38]. NAS can behave as commensal, opportunistic, and obligate pathogenic microorganisms [39], and the variety of NAS species contributes to the diversity of the epidemiological findings [40]. NAS are unique, as some are commensal organisms of the teat canal and others may result in IMI. The commensal nature of NAS presents a considerable risk in terms of AMR selection [41]. It has been suggested that NAS could act as a possible reservoir for resistance genes [42] and that these genes could contribute to the spread of AMR [41]. Therefore, we believed the risk of NAS causing infection is lower than the risk of NAS in terms of AMR selection, which highlights the fact that it is not only cows that are screened as negative for mastitis at drying-off that can be managed with ITS alone but also cows with lower-SCC NAS mastitis-causing levels.

The majority of dairy herds has adopted the practice of blanket DCT [3]. However, the use of antibiotics in all cows at drying-off includes healthy cows, which ends up being a prophylactic treatment. A recommended blanket DCT has been a strategy adopted in some countries (e.g., Brazil), while in others, such as the Netherlands, producers need to select only those cows at risk of mastitis for drying off using antibiotics (MARAN, 2018). In our study, culture-negative results accounted for > 85% of the mammary quarters being considered healthy, in which there was no isolation of pathogen causing the disease and no elevated SCC. In this case, we can infer that the indiscriminate use of antibiotics would have occurred in the farm enrolled in this current study if SDCT had not been adopted. In other words, antibiotic use should be prioritized for animals at risk, thereby potentially reducing antimicrobial metaphylactic use.

Inferences have been made about how the use of blanket DCT can upset the microbiome of a healthy mammary gland [11]. In fact, DCT could affect the beneficial bacteria of the mammary gland directly. Furthermore, the findings of studies using a bacterial DNA-based methodology have called into question the concept of the sterility of the healthy mammary gland of dairy ruminants [17], since the existence of commensal microbial communities was reported. Thus, mastitis has been considered a pathogen-host interaction by some researchers and considered to be dysbiosis: that is, an imbalance in the microbiota of the mammary gland [17,18,41,43]. On the other hand, Rainard [44] stated that the existence of an intramammary microbiota is a myth that could lead to confusion and may interfere with effective mastitis control procedures. In our study, the inclusion of ATB did not shift bacterial diversity, as similarly reported in a previous study [11]. Furthermore, we speculate that if ATB induces disturbances in the milk microbiome, it would be minor, being turned around by the start of a new lactation process since microbial communities are dynamic, as described in a previous study [11].

A similar prevalence of NIMI was observed in healthy cows treated by DCT or SDCT [7]. Therefore, healthy mammary quarters do not seem to be protected by ATB usage. Our results showed that the estimated NIMI risk, as ascertained by logistic regression analysis, was 10 cases per 100 quarters at risk using ATB + ITS, and 7 when using ITS alone (data not shown). Similarly, in comparison with untreated quarters, the use of ITS was related to a lower number of NIMI in the post-calving period and the development of clinical mastitis in the first 100 days following lactation [15]. Furthermore, Rabiee and Lean [12] described an average 25% reduction in NIMI risk when using DCT but a 73% reduction in NIMI risk when using only ITS in healthy quarters.

The number of observed ASVs, Shannon’s evenness, and the inverse of the Simpson diversity index were not significantly different among mammary quarters receiving ATB + ITS or ITS alone. Our results were similar to those described in a previous study from Bonsaglia [11] but did not corroborate those reported by other researchers [5], which found variations in alpha diversity within 14 days in cows with experimentally induced mastitis following ATB treatment. This could be attributed to the fact that Bonsaglia [11] included only healthy cows in the experimental design, but Ganda [5] included both healthy and mastitic cows. It is noteworthy that we did not observe variations in alpha diversity when comparing healthy versus infected cows, a fact that can be explained by the majority of healthy mammary quarters found. Furthermore, in the present study, treatment, the time of sampling, or the interaction of these factors did not affect the bacterial community in the milk samples. Similar to the beta-diversity analysis results found by Bonsaglia [11], our results showed that there is not a clear distinction in structure/composition in bacterial communities between healthy and cured groups.

Microbiota composition can vary in terms of the different niches of the udder [45]. Even though it is well known that the teat canal and teat skin have commensal microbiota, it has been hard to show that the mammary gland has a commensal core-microbiota because of methodological problems and a lack of consistency [46]. Within the microbiota of healthy cows maintained in indoor free-stall barns, *Corynebacterium, Ruminococcus, Aerococcus, Bifidobacterium,* and *Facklamia* were prominent bacterial genera within the microbiota of the teat cistern and teat canal. However, *Staphylococcus* spp., particularly NAS species, appear to be among the most common colonizers, even when cows are subjected to pre- and post-milking teat disinfection. Notwithstanding that we did not focus on cisternal and teat canal microbiota, we observed an abundance of some pathogens from these niches in the microbiota of milk samples (e.g., NAS).

**Figure 4:**
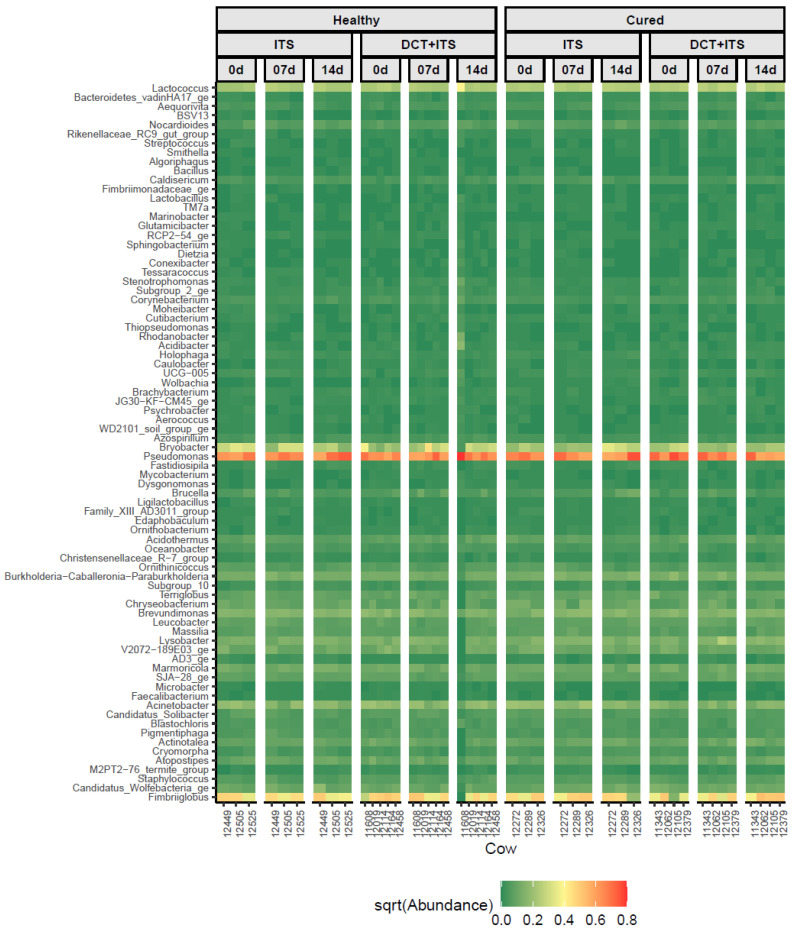
Distribution of the most abundant bacterial genera (*n* = 78) that were present in at least 50% of all samples, among individual milk samples from cows according to category (Healthy and Cured), treatment (ITS and DCT+ITS), and time of sampling (0 d: the day of drying-off, 7 d: 7 days after calving, and 14 d: 14 days after calving). The color key represents the square root transformed (sqrt) relative abundance according to the gradient of color, from dark green (low abundance) to dark red (high abundance).

The microbial composition of milk samples collected from healthy quarters has not been uniform throughout the studies cited [45]. Even while bacterial genomes from milk, teat skin, and the teat canal are commonly detected, environmental factors have been demonstrated to greatly affect the composition of these bacterial populations; therefore, it is unclear whether these organisms are viable, where they came from, or what they do [47]. When the microbiota of milk samples from clinically healthy quarters and those from culture-negative mastitic quarters were compared, the latter group had higher proportions of the genera *Burkholderia, Sphingomonas,* and *Stenotrophomonas,* whereas the microbiota of healthy quarters had an overrepresentation of the genera *Pseudomonas, Psychrobacter,* and *Ralstonia* [48]. In this current research, we did not find differences in bacterial diversity ascribed to category and treatment. However, the most prevalent bacterial genera in the microbiota of milk samples was found to have a similar distribution [45], as shown in the heatmap in Figure 4. Likewise, common bacterial genera, such as *Acidobacter, Acinetobacter, Aerococcus, Bacillus, Bacteriodetes, Brevibacterium, Brevundimonas, Bryobacter, Burkholderia, Corynebacterium, Fimbriiglobus, Lactobacillus, Lactococcus, Lysobacter, Microbacter, Nocardioides, Pseudomonas, Psychrobacter, Staphylococcus, Stenotrophomonas, Sphingobacterium,* and *Streptococcus,* were observed as being similar to those described in previous studies [11,18,43,44,47,48]. Proteobacteria was the most abundant phylum in our study, which differed from that observed in previous studies [11,45] wherein Firmicutes was the most abundant phylum. In general, Firmicutes, Proteobacteria, Bacteroidetes, and Actinobacteria have been the most abundant and prevalent phyla, which finding was similar to our results.

Interestingly, our differential abundance analyses showed that the abundance of several bacteria genera increases or decreases when using ATB (DCT+ITS), in comparison to the use of ITS alone. However, to our knowledge, neither of the genera found in this analysis is a real cause of mastitis. Briefly, we showed that quarters treated with ATB (DCT+ITS) and categorized as cured had an increase in *Microbacter* at 7 days and *Bacillus* at 14 days after calving; conversely, quarters treated with ATB (DCT+ITS), but categorized as healthy had a decrease in *Prevotella* at 7 days and *Methyloversatilis* and *Acholeplasma* at 14 days after calving, in comparison to quarters treated with ITS alone. Further research is warranted to investigate the effect of ATB-based treatments on the fastidious microorganisms that can be isolated from milk samples. There are still many gaps related to the roles of these uncertain pathogens concerning the mammary gland. We speculate that the indiscriminate use of ATB in healthy quarters at drying-off can increase the risk of infection from environmental mastitis-causing pathogens in the next lactation. Thus, the use of ITS alone in healthy cows would support a favorable environment for those unknown pathogens of the milk microbiome, helping microbiome stability during the drying-off [11].

Cow characteristics, such as parity, lactation stage, season, or any other herd-level descriptor, can influence the risk of mastitis. Mammary gland infection is more common among older cows and in those that are in the early stages of lactation, as well as during the summer months [49]. Furthermore, the microbiome composition is also linked to these influences [47]. The current study did not consider these factors to evaluate the association with microbiota composition and it can be considered a limitation.

It is important to understand how the quarter milk microbiota works, so that effective treatment plans can be made and a healthy microbiota physiology can be restored. The adoption of ATB-based protocols for drying off only in cows with mastitis is essential to implementing programs that try to manage dairy cows with the use of fewer ATB. With this in mind, it is the opinion of the authors that banning the sale of ATBs without a prescription and ending the prophylactic use of ATB (e.g., to encourage the growth of healthy animals) are two important measures to avoid the non-prudent use of antibiotics in veterinary medicine. These are two policies in force in the European Union, but they are unimaginable in those countries where blanket DCT has been recommended.

## 5. Conclusions

Healthy cows treated at drying-off using only teat sealant showed no alteration in the alpha and beta diversity but showed a higher abundance of those bacterial groups that may be beneficial to or commensals of the mammary gland. Our preliminary results suggest that the use of blanket dry cow therapy should be recommended only for those cows with a history of subclinical or clinical mastitis.

## Figures and Tables

**Table 1:** Culture results (day 0: the drying-off day, and on days 7 and 14 post-calving), on-farm established groups for the adoption of drying-off protocols, and category definitions.

Cow Identification	Treatments ^1^	Culture Results on the Day of Drying-Off	Culture Results at Post-Calving		Categories
		Day 0	Day 7	Day 14	
12272	ITS alone	*Staphylococcus haemolyticus*	Negative	Negative	Cured ^2^
12326	ITS alone	*Staphylococcus haemolyticus*	Negative	Negative	Cured
12289	ITS alone	*Staphylococcus haemolyticus*	Negative	Negative	Cured
11747	ITS alone	*Staphylococcus simulans*	*Staphylococcus simulans*	*Staphylococcus simulans*	Persistent ^3^
12525	ITS alone	Negative	Negative	Negative	Healthy ^4^
12440	ITS alone	Negative	Negative	*Aecococcus viridans*	NIMI ^5^
12505	ITS alone	Negative	Negative	Negative	Healthy
12441	ITS alone	Negative	Negative	*Aerococcus viridans*	NIMI
12449	ITS alone	Negative	Negative	Negative	Healthy
12399	ITS alone	Negative	*Staphylococcus simulans*	*Staphylococcus simulans*	NIMI
12379	DCT + ITS	*Lactococcus garvieae*	Negative	Negative	Cured
12324	DCT + ITS	Negative	*Aerococcus viridans*	Negative	NIMI
11343	DCT + ITS	Negative	Negative	Negative	Cured
12062	DCT + ITS	Negative	Negative	Negative	Cured
12105	DCT + ITS	Negative	Negative	Negative	Cured
11608	DCT + ITS	Negative	Negative	Negative	Healthy
12114	DCT + ITS	Negative	*Acinetobacter townen*	Negative	Healthy
12164	DCT + ITS	Negative	Negative	Negative	Healthy
12458	DCT + ITS	Negative	Negative	Negative	Healthy
12019	DCT + ITS	Negative	Negative	Negative	Healthy

^1^ ITS: only internal teat sealant was used when the four quarters of a cow had no isolation of mastitis-causing agents, no history of clinical mastitis, and the last three monthly SCC values were < 200 × 10^3^ cells/mL; DCT + ITS: antibiotic and sealant was used when a cow was culture-positive in at least one quarter and at least one of the three monthly SCC > 200 × 10^3^ cells/mL; ^2^ Cured: a positive isolation result at the day of drying-off but a negative result after calving; ^3^ Persistent: when there was isolation of the same agent at the day of drying-off and after calving; ^4^ Healthy: no isolation of microorganisms in all microbiological culture results; and ^5^ New infection: when there was no isolation at the day of drying-off and there was an isolation after calving, or when there was isolation in the post-calving sampling of microorganisms that differed from the agent isolated at the day of drying-off.

**Table 2:** Summary of sequencing of bacterial 16S rRNA of milk samples, grouped according to category, treatment, and time point.

Treatment	Category	Time	*n*	Sequences after Normalization		
				Total	Mean	SD
ITS ^1^	Healthy ^3^	0 d	3	22,262.00	7420.67	201.10
		7 d	3	22,326.00	7442.00	199.73
		14 d	3	21,801.00	7267.00	351.10
	Cured ^4^	0 d	3	22,246.00	7415.33	205.51
		7 d	3	22,058.00	7352.67	94.04
		14 d	3	21,715.00	7238.33	253.68
	Persistent ^5^	0 d	1	6808.00	6808.00	0.00
		7 d	1	7306.00	7306.00	0.00
		14 d	1	9309.00	9309.00	0.00
	New infection ^6^	0 d	3	21,905.00	7301.67	68.07
		7 d	3	21,639.00	7213.00	582.29
		14 d	3	21,590.00	7196.67	294.98
DCT ^2^ + ITS	Healthy	0 d	5	36,906.00	7381.20	237.57
		7 d	5	36,623.00	7324.60	172.23
		14 d	5	36,479.00	7295.80	467.70
	Cured	0 d	4	28,734.00	7183.50	201.02
		7 d	4	28,256.00	7064.00	253.70
		14 d	4	28,430.00	7107.50	170.77
	Persistent	0 d	1	6984.00	6984.00	0.00
		7 d	1	7292.00	7292.00	0.00
		14 d	1	5757.00	5757.00	0.00

^1^ Internal teat sealant; ^2^ dry cow therapy; ^3^ Healthy: no isolation of microorganisms in all microbiological culture results; ^4^ Cured: a positive isolation result on the day of drying-off but a negative result after calving, or if the microbiological culture result after calving differed from the one at the day of drying-off; ^5^ Persistent: when there was the isolation of the same agent at the day of drying-off and after calving; ^6^ New infection: when there was no isolation at the day of drying-off and there was isolation after calving or when there was isolation in the post-calving sampling of microorganisms that differed from the agent isolated at the day of drying-off.

**Table 3:** Summary of analysis of variance of the bacterial alpha diversity of milk samples.

Category	Index	Factor	numDF	denDF	F. Value	*p*-Value	Padj (Method = BH Aka FDR ^1^)
Healthy ^2^	Sobs ^4^	Treatment	1	6	0.316	0.595	0.649
		Time	2	12	3.080	0.083	0.146
		Treatment:Time	2	12	2.883	0.095	0.146
	InvSimpson ^5^	Treatment	1	6	4.740	0.072	0.146
		Time	2	12	0.350	0.712	0.712
		Treatment:Time	2	12	0.555	0.588	0.649
	Shannon ^6^	Treatment	1	6	14.556	0.009	0.026
		Time	2	12	0.681	0.525	0.649
		Treatment:Time	2	12	2.851	0.097	0.146
Cured ^3^	Sobs	Treatment	1	5	0.519	0.503	0.503
		Time	2	10	4.729	0.036	0.072
		Treatment:Time	2	10	15.282	0.001	0.002
	InvSimpson	Treatment	1	5	95.276	0.000	0.001
		Time	2	10	2.240	0.157	0.236
		Treatment:Time	2	10	1.875	0.203	0.244
	Shannon	Treatment	1	5	1.649	0.255	0.279
		Time	2	10	2.802	0.108	0.185
		Treatment:Time	2	10	2.004	0.185	0.244

^1^ False discovery rate (FDR) aka Benjamini–Hochberg (BH) adjusted *p*-value; ^2^ Healthy: no isolation of microorganisms in all microbiological culture results; ^3^ Cured: a positive isolation result on the day of drying-off but a negative result after calving; ^4^ number of observed ASVs; ^5^ Shannon’s evenness; ^6^ inverse of the Simpson diversity index.

**Table 4:** Summary of pairwise comparisons (least-squares means) of the alpha diversity indices of milk bacterial communities.

Category	Index	Factor	Pairwise	Ratio	SE	df	Null	t.Ratio	*p*-Value
Healthy ^1^	Shannon ^3^	Treatment	ITS vs. DCT + ITS	1.00	0.00	6.00	1.00	−0.91	0.40
Cured ^2^	Sobs ^4^	Treatment * Time ^5^	ITS 0 d vs. DCT + ITS 0 d	1.20	0.08	5.00	1.00	2.81	0.20
			ITS 0 d vs. ITS 7 d	1.15	0.06	10.00	1.00	2.57	0.19
			ITS 0 d vs. DCT + ITS 7 d	0.99	0.06	5.00	1.00	−0.11	1.00
			ITS 0 d vs. ITS 14 d	1.21	0.10	10.00	1.00	2.30	0.28
			ITS 0 d vs. DCT + ITS 14 d	1.14	0.09	5.00	1.00	1.67	0.60
			DCT+ITS 0 d vs. ITS 7 d	0.95	0.06	5.00	1.00	−0.80	0.95
			DCT+ITS 0 d vs. DCT + ITS 7 d	0.83	0.04	10.00	1.00	−4.11	0.02
			DCT+ITS 0 d vs. ITS 14 d	1.01	0.08	5.00	1.00	0.08	1.00
			DCT+ITS 0 d vs. DCT + ITS 14 d	0.95	0.07	10.00	1.00	−0.76	0.97
			ITS 07 d vs. DCT + ITS 7 d	0.86	0.04	5.00	1.00	2.80	0.20
	InvSimpson ^5^	Treatment	ITS vs. DCT + ITS	0.96	0.10	5.00	1.00	−0.38	0.72

^1^ Healthy: no isolation of microorganisms in all microbiological culture results; ^2^ Cured: a positive isolation result on the day of drying-off but negative result after calving; ^3^ Number of observed ASVs; ^4^ Shannon’s evenness; ^5^ Inverse of the Simpson diversity index; * Interaction term between Treatment and Time.

**Table 5:** Beta diversity analysis: summary of the permutational multivariate analysis of variance (PERMANOVA nperm = 1000) of Bray–Curtis dissimilarities in the bacterial community of milk samples.

Category	Factor	Df	SumsOfSqs	MeanSqs	F.Model	R2	Pr (>F)
Healthy ^1^	Treatment	1	0.01	0.01	0.49	0.02	0.97
	Time	2	0.05	0.02	0.78	0.07	0.75
	Treatment * Time ^3^	2	0.06	0.03	1.06	0.10	0.34
	Residuals	17	0.49	0.03	-	0.80	-
	Total	22	0.61	-	-	1.00	-
Cured ^2^	Treatment	1	0.04	0.04	1.39	0.07	0.15
	Time	2	0.06	0.03	1.01	0.10	0.41
	Treatment * Time	2	0.05	0.03	0.90	0.09	0.58
	Residuals	15	0.46	0.03	-	0.74	-
	Total	20	0.62	-	-	1.00	-

^1^ Healthy: no isolation of microorganisms in all microbiological culture results; ^2^ Cured: a positive isolation result on the day of drying-off but a negative result after calving. ^3,^* Interaction term between Treatment and Time.

## Data Availability

All DNA sequences have been submitted to the NCBI’s Sequence Read Archive as part of BioProject PRJNA776327.
